# A review of causal inference in forensic medicine

**DOI:** 10.1007/s12024-020-00220-9

**Published:** 2020-03-10

**Authors:** Putri Dianita Ika Meilia, Michael D. Freeman, Herkutanto, Maurice P. Zeegers

**Affiliations:** 1 Care and Public Health Research Institute (CAPHRI), Maastricht University Medical Center+, Universiteitssingel 40, 6229 ER Maastricht, the Netherlands; 2 Department of Forensic Medicine and Medicolegal Studies, Faculty of Medicine, University of Indonesia, Jl. Salemba Raya No. 4, Salemba, Jakarta Pusat, 10430 Indonesia

**Keywords:** Causality, Causal inference, Intuitive causation, Probabilistic causation, Forensic medicine

## Abstract

The primary aim of forensic medical analysis is to provide legal factfinders with evidence regarding the causal relationship between an alleged action and a harmful outcome. Despite existing guides and manuals, the approach to formulating opinions on medicolegal causal inference used by forensic medical practitioners, and how the strength of the opinion is quantified, is mostly lacking in an evidence-based or systematically reproducible framework. In the present review, we discuss the literature describing existing methods of causal inference in forensic medicine, especially in relation to the formulation of expert opinions in legal proceedings, and their strengths and limitations. Causal inference in forensic medicine is unique and different from the process of establishing a diagnosis in clinical medicine. Because of a lack of tangibility inherent in causal analysis, even the term “cause” can have inconsistent meaning when used by different practitioners examining the same evidence. Currently, there exists no universally applied systematic methodology for formulating and assessing causality in forensic medical expert opinions. Existing approaches to causation in forensic medicine generally fall into two categories: intuitive and probabilistic. The propriety of each approach depends on the individual facts of an investigated injury, disease, or death. We opine that in most forensic medical settings, probabilistic causation is the most suitable for use and readily applicable. Forensic medical practitioners need, however, be aware of the appropriate approach to causation for different types of cases with varying degrees of complexity.

## Introduction

Forensic medicine (FM) refers to the discipline concerned with the application of medical knowledge and technology in legal proceedings [1–3]. Although the practice, definitions, and applications of FM vary widely by geographic and jurisdictional region [4], the most common primary aim of forensic medical analysis is to provide legal factfinders (i.e. judge or jury) with evidence regarding the causal relationship between an action allegedly committed by the accused (in a criminal matter) or defendant (in a civil matter) and a medically observed harmful outcome (injury, disease, or death). The results of a causal analysis are usually presented to the court in the form of an expert opinion. A causal analysis opinion may address general causation (whether an exposure *can* cause the injury observed in an individual) or specific causation (whether the exposure *did* cause the injury observed in the individual), or both. Expert opinions regarding causal inference, especially specific causation, is an essential element in most legal actions associated with an injury, as they provide evidence of the connection between an alleged unlawful or negligent act committed by one party and an observed adverse health effect in another [5].

Despite existing guides and manuals [6–8], the approach to formulating opinions on medicolegal causal inference used by forensic medical practitioners, and how the strength of the opinion is quantified, remains an element of FM practice that is mostly lacking in an evidence-based or systematically reproducible method. In the practice of medicolegal autopsy, for example, cause-of-death determination is often performed by subjectively appointing *a* cause of death found at the autopsy as *the* cause of death, making the determination idiosyncratic to the experience and personal thought processes of the individual clinician and potentially irreproducible by other similarly experienced clinicians [9, 10]. In other circumstances encountered in an FM analysis, the causal assessment may be more challenging because of either insufficiency of the quantify or reliability of physical evidence or, conversely, a surfeit of findings with manifold and even mutually exclusive interpretations [11]. In order to move in the direction of both valid and repeatable causal conclusions, a technically correct and sufficiently complete forensic examination of evidence must be combined with the application of generally accepted causal methods.

In the present review, we sought to define and discuss the literature describing existing methods and approaches of causal inference used by forensic medical practitioners, especially in relation to the formulation of expert opinions in legal proceedings. The strengths and limitations of the various causal approaches identified in the review are also presented.

## Cause, causal relationship, and causation

Forensic medical practitioners provide opinions for fact-finders regarding the cause of death, injury, or disease on a routine basis, yet because the concept of causation is somewhat abstract (particularly when compared with other more widely-known medical concepts, such as “diagnosis”) the definition of what constitutes a cause is somewhat esoteric. The history of the study of injury and disease causality is tortuous and interdisciplinary, crossing from philosophy into science and medicine, and ultimately finding a home primarily in epidemiology. Many pre-eminent scientists and philosophers of their day, including Galileo Galilei, Karl Popper, David Hume, Bertrand Russell, Kenneth Rothman, John Stuart Mill, Robert Koch, and Austin Bradford Hill, have described various definitions of what a cause is, and/or how to demonstrate that a causal relationship is present [13].

In the current era, a cause is often defined as an antecedent event or condition which is necessary for the manifestation of the effect at the moment it occurred. Without the cause, the effect either would not have occurred at all or would have occurred later, given that other conditions are fixed [12]. Notwithstanding this simple definition, a cause can also be deemed as an exposure that only increases the probability of an effect [13, 14]. There are three common attributes or properties of a cause, i.e. association (meaning that the effect occurs more often among those who are exposed than those who are not exposed), temporal order, and asymmetric direction of change [13].

A definition that is often used in medicolegal investigations of causation is that of the probabilistic cause (i.e. an antecedent exposure [A] which increases the probability of the occurrence of an effect [B]) in a counterfactual setting. A counterfactual is a hypothetical scenario that is contrary to the fact or reality, i.e. what would have happened if a different exposure had occurred (A’ instead of A, everything else being constant) [15]. There are 4 types of (causal) relationships that may exist between A and B, which are not necessarily mutually exclusive, as follows: (1) A causes B, (2) B causes A, (3) A and B have a common cause, and (4) A and B are not causally related [16].

The main difficulty in establishing causation is that causal relationships are unobservable; past events can no longer be observed [17]. Causation can only ever be known through inductive inference; it can never be directly demonstrated [18]. What we can do is evaluate the attributes of a potential cause or assess specific criteria of causation. Causation is always a judgment; even when we think that a causal relationship exists, there is no guarantee that we are right [14]. All existing models/approaches to causal evaluation are to a certain degree based in Hill’s viewpoints, which were designed to assess the causal relationship of observed associations in populations, in part to determine if they are overtly spurious or not [19]. Temporal sequence is the only causal element that must be present in all cases. This relationship is, however, unidirectional; as the presence of an appropriate temporal sequence only demonstrates coincidence when a causal relationship is deemed implausible according to known scientific principles.

## Causal inference

Causal inference is a unique type of scientific reasoning; there are no fixed set of causal criteria which can establish the validity of an inference [12]. It is a scientific judgement about the probability of a particular hypothesis based on the evaluation and weighting of various types of evidence [20]. Currently, causal inference is not part of the formal medical curriculum [21] because it is commonly considered as an innate ability of doctors to form logical conclusions based on their examinations. Actually, it is an acquired skill [22], which requires sufficient (medical) background knowledge and a lot of practice.

There are three categories of approach to probabilistic inference, i.e. forecasting, back-casting, and attribution [23]. Forecasting is the probability of an outcome given a particular exposure, whereas back-casting is the probability of an exposure given a specific outcome. Both are conditional statements and are sometimes referred to as “Effects of Causes (EoC),” and both are commonly used in medical science, particularly epidemiology. Attribution, on the other hand, is the probability that the outcome would not have occurred if it were not for a particular exposure. As such, attribution is a counterfactual statement and is referred to as “Causes of Effects (CoE).”

A counterfactual is a hypothesis of what would have happened under conditions which are contrary to actual conditions [24]. In other words, we try to envision what would the outcome be if B happened instead of A. We specify the counterfactual world by altering the suspected cause and then compare it to the factual circumstances [25]. For example, would the driver in a head-to-head car collision have died at that moment if he had worn a seatbelt? In assessing counterfactuals, we need to specify sufficiently-well defined versions of the potential outcome and its counterfactual contrast, given the circumstances, by taking all available knowledge/evidence into account [26–30] and assuming a ceteris paribus condition, i.e. all other things or conditions being equal [31]. Because a counterfactual condition cannot be observed directly, and counterfactual outcomes cannot be determined in an individual, we need a substitute from which we can infer it. This substitute can either be a different target population or a different period [24, 32].

## General versus specific/individual causation

The basic steps of causation analysis consist of determining general (‘can-it’ and ‘does-it’) and specific/individual (‘did-it’) causation [33]. Any individual causal analysis must consider general causation before trying to answer the question of specific causation, i.e. ‘ruling in’ the alleged causal agent before ‘ruling out’ other possible causes [34]. General causation is typically addressed at a population or pathophysiological level. This means that the plausibility of the relationship is established either via epidemiologic studies demonstrating an increased risk of injury from the exposure, or the injury is plausibly associated with the exposure despite the lack of epidemiologic evidence. The latter relationship may be assessed via some of the Hill criteria that address the plausibility of a causal relationship (e.g. coherence, analogy, specificity, etc.). Specific (individual) causation addresses whether the specific exposure was the cause of the diagnosed injury or disease in is the subject of discussion in many legal proceedings [18].

Specific causation is drawn from group-to-individual (G2i) inference based on the following assumptions: the risk estimate comes from a valid study, the individual is sufficiently similar to the study subjects with respect to important risk factors, the putative cause does not cause acceleration of outcome or provide protective effect and operates independently [35, 36]. G2i inference can be justified if the individual possesses no features which make him/her more or less susceptible to the putative exposure’s ability to cause the outcome in question than other members of the population/reference group. In other words, the affected individual must be comparable with regard to critical, and potentially confounding, characteristics, and can thus be treated as randomly selected from the population [18, 37–40].

## Probability and the probability of causation (PC)

Probability reflects the degree of (un)certainty or conviction of the truth of an assertion for a given set of facts (conditional probability). Probabilistic causation uses Bayesian reasoning, which tells us what we want to know given what we do know, which are known as its ‘conditions’ and can be a combination of data (objective probability) and experience or knowledge (subjective probability) [41–43]. To obtain a correct value of probability, we must avoid vagueness in the causal analysis by clearly defining the terms and the relationships between the terms in the causal model [44].

If we want to express probabilities in words, we must ensure that we use the same words for the same values consistently. As an effort to clarify the meaning of probabilities in court, forensic scientists have often used numbers (quantitative explanation) followed by a verbal/qualitative interpretation [42, 45]. For example, a PC of >50% is often stated as “more likely than not,” a PC of >98% is sometimes used to indicate that something is “beyond reasonable doubt,” or using the term “to a reasonable degree of scientific certainty” to convey the general impression of confidence regarding their expert opinion. This method is, however, not without problems. Besides having no scientific meaning, those terms might mislead legal factfinders about the level of objectivity involved in the analysis, as well as its reliability and limitations in reaching an individualized conclusion [46].

Probabilities can be compared in different ways, e.g. by calculating the difference between two incidences (i.e. the risk difference (RD) or attributable risk (AR)) or by dividing them (i.e. the relative risk or risk ratio (RR)) [41]. The likelihood ratio is a ratio of two probabilities, i.e. the probability of the evidence supposing one hypothesis is true divided by the probability of the evidence if an alternative hypothesis is correct. The likelihood ratio can only be useful in comparing two hypotheses if they are mutually exclusive and reflect the opposing standpoints of the hypotheses [42].

The probability of causation (PC) expresses the amount of causation attributable to a component cause, which is equal to the reduction of the risk of the condition in a population that is not exposed to the cause. It should never be interpreted as the probability that a putative cause is the single cause of the effect in an individual [30]. There are dissenting opinions about the bounds of the PC in specific causation. First, some opine that epidemiological evidence can be used to estimate a lower bound on the probability of causation (PC: PC ≥ 1 – (1/RR)). The PC can then be used to fulfil the but-for test on the balance of probabilities [18]. The but-for test itself can be described in simple terms as follows: but-for the exposure or putative cause, would the individual still have the disease or experienced the injury at the same time, everything else being constant? The test, therefore, basically asks a counterfactual question, which is a hypothetical scenario that is contrary to the fact or reality. Another opinion is that inference about CoE from epidemiological data is usually presented as a PC with upper and lower bounds. As such, it contains triple uncertainties, i.e. it is a probability with interval bounds, which are imprecise themselves [47]. Yet another opinion is that because the PC itself is the degree of (un-)certainty in considering the defined counterfactual conditions based on his/her present knowledge of the facts it is unnecessary to add bounds to it, which will only add uncertainties to an uncertainty. If there is new knowledge, the PC can be adjusted to reflect the current state of belief [48].

## The role of forensic medical expert opinions in legal causal analysis

Causal analysis plays a vital part in establishing liability [5]. There are two aspects of causation, i.e. factual/cause-in-fact causation and proximate/legal causation [12, 49]. The role of forensic medical expert opinions in legal proceedings is to help determine factual causation by assessing probabilities for the “but-for” test, which are then used by the legal fact-finders to establish a proximate cause in the form of proportional liabilities [6, 50–52]. An essential role of the forensic medical expert is to quantify the PC, which denotes the probability that the defendant’s act caused the effect in the claimant [53]. A primary obstacle in drawing causal inferences is the binary format of the legal question for causation. It is based on the ‘but-for’ test and demands a simple yes or no answer [30].

The most important principle to be remembered is that expert opinions must be useful to the legal fact finder. Thus, they must be applicable to the individual case at hand [54]. Expert opinions should be informative, or at least not be misleading and/or speculative [34]. A helpful forensic medical expert opinion has several features: (1) it provides a concise and straightforward answer, (2) it states the reasons or justifications for a particular opinion which can be upheld in court, (3) in cases where it is impossible to provide a definite answer, it gives the best possible explanation on a ‘balance of probabilities’, and (4) it must be clearly and appropriately worded to prevent any ambiguities [6, 40, 42, 55].

Often, courts must rely on expert testimony that is not much more than an experience-based clinical judgment. When there is a difference in opinion between experts, the legal fact finder should be able to evaluate whether those opinions are based on valid and rational approaches. Therefore, any methods used by an expert should be transparent and justifiable based on the best available scientific evidence [33, 56]. It is perfectly acceptable for experts to base their claims on assumptions if those assumptions are made transparent. In that way, others can judge whether they are plausible [57]. The Daubert principle might be considered as demand for ‘evidence-based (expert) evidence’, as it asks judges to act as gatekeepers by ensuring that (forensic) expert evidence to be admitted in court is not only relevant but also reliable [58, 59]. This can be achieved through the development of ‘clinical practice guidelines’ in formulating expert opinions [59].

## Existing medicolegal causal analysis approaches

Medicolegal causal analysis is the process of determining the nature and probability of a relationship between a putative cause and a claimed effect. Generally, the complex process of medicolegal causal analysis is poorly conceptualized, not standardized, and only supported by the expertise or experience of the individual expert [60]. Currently, there exist several approaches to medicolegal causal analysis in forensic medicine. All approaches generally fall into two categories, i.e. either they are intuitive or probabilistic. The presence of Hill’s viewpoints marked the transition from the purely intuitive approaches to the more systematic, analytic approaches, which then led to the conception of the probabilistic ones. Table 1 summarizes those approaches, their strengths and weaknesses, as well as the type of cases for which they are appropriate.

**Table 1:** Existing approaches to medicolegal causal analysis

No.	Approach	Category	Application	Example	Strengths	Weaknesses
1.	Intuitive approach (i.e. scientific common-sense)	Intuitive	Simple cases where the causal relationship “makes sense” based on fundamental scientific principles	Death following a gunshot wound to the head	Practical, does not need exceptional/additional resources	Not suitable for more complex cases where the causal relationship is not as readily apparent
2.	Categorical intuitive deduction (i.e. the Sherlock Holmes style or educated guess)	Intuitive	Cases where there is only one plausible cause at the same time	Death following ingestion of insecticides in a previously healthy person with no signs of trauma	Impressive expert witness testimony	Not suitable for more complex cases where there is more than one plausible cause, requires a lot of professional experience, potentially misleading
3.	Hill’s viewpoints [19]	Intuitive-probabilistic	Cases with sufficient epidemiologic evidence and literature to assess competing causal hypotheses	Post-traumatic headache in a sexual-assault victim	Check-list-like criteria to guide causal inference	Temporal sequence is the only real “causal criterion” [76], the meaning or value of the other criteria can be unclear [77]
4.	The American Medical Association (AMA) Guides to the Evaluation of Disease and Injury Causation [6, 61]	Intuitive-probabilistic	Primarily cases of injury with multiple plausible causes and work-related conditions	Lower back-pain in a factory worker who stands all-day	Provide elements that may be used for a systematic step-by-step causal analysis, primarily to assess work-relatedness	Lengthy and complicated process, does not produce a PC/quantification of the level of certainty
5.	Forcier-Lacerte medicolegal causal analysis model [60]	Intuitive-probabilistic	Primarily cases of injury with multiple plausible causes and cases related to insurance claims	An elderly woman with severe osteoporosis who sustains a slip-and-fall resulting in several fractured ribs	Provide elements that may be used for a systematic step-by-step causal analysis, categorizes possible causes into (1) the accident, (2) preexisting health status, and (3) intervening event.	Lengthy and complicated process, does not produce a PC/quantification of the level of certainty
6.	The epidemiology-based approach by Siegerink et al. [30]	Probabilistic	Civil litigations or cases of tort, where the issue is primarily about the proportional liability of multiple plausible causes	The risk of lung cancer in a factory worker exposed to asbestos, who is also a heavy smoker with a family history of lung cancer	Fits both the sufficient cause model and the counterfactual model, offers proportional liability for each component cause (i.e. the unlawful act plus other possible factors)	Could overestimate the number of components of the sufficient cause, leading to an underestimation of liability, all components are considered as of equal importance, while from a legal perspective some causes may be more important than others (e.g. unlawful act vs genetics)
7.	The 3-step medicolegal causation approach by Freeman [8]	Probabilistic	Cases of injuries with multiple plausible causes that do not require a high degree of energy, preexisting conditions which only become symptomatic after relatively minor trauma, or conditions with an insidious symptom onset	An elderly woman with shoulder pain after a minimal-damage rear-impact collision	Practicable, systematic, fits the standards of both medical and legal practice by establishing (1) plausibility, (2) temporality, and (3) the absence of a more probable alternative explanation (differential etiology)	Requires sufficient epidemiologic data and comprehension of epidemiologic methods to compare risks of differential etiologies
8.	The forensic epidemiology approach [62, 63]	Probabilistic	Highly complex cases with multiple plausible causes	Peripartum cardiomyopathy in a young woman following exposure to doxorubicin	Systematic, provides a scientifically valid and verifiable quantification of probability in the form of a comparative risk ratio (CRR) and a probability of causation (PC), results are suitable for presentation in a court of law	Uses epidemiologic principles, methods, and data to formulate a probability, the analyses and calculations can be quite complicated, might not be suitable for day-to-day forensic medical practice

## Discussion

Causal inference in forensic medicine is different from the process of establishing a diagnosis in clinical medicine. Diagnoses in clinical medicine are prospective in nature; i.e. they move from diagnosis to a specific clinical decision (e.g. a treatment) and, thus, have an empirical aspect allowing for observation of and patient feedback on the efficacy of the strategy, and thereby the correctness of the diagnosis. Gold standards are often available for clinical practice, (an example is an autopsy to confirm the presence of antemortem diagnosis), and the use of such standards leads to broadly accepted standards for diagnosis and treatment. In contrast with clinical medicine, forensic medical investigations are all conducted retrospectively. One of the central goals of a forensic medical investigation is to assess the causal relationship between a particular action of interest and an adverse injury or disease health event that has occurred prior to the investigation. As causal investigations are always retrospective, the cause of an injury or disease can never be observed or measured directly (like a diagnosis). An additional facet of causal investigation in forensic medicine is that there are no actual gold standards, unlike in clinical medicine, against which one can ascertain the validity of a causal inference [9]. Whether or not a causal relationship is present between an investigated action and a subsequently observed disease or injury, as well as the strength of the relationship, must be determined inferentially, via weighing and comparing of risks [62].

In addition to the inherent subjectivity of causation opinions, several additional factors threaten the validity and reliability of forensic medical expert opinions. First, forensic medical practitioners tend to rely more on experience and individual customary practice in formulating their opinion than on evidence or consensus-based practices [64, 65]. Additionally, the strategy used in formulating expert opinions varies substantially between experts [66, 67]. Expert inferential opinions, particularly those regarding causation, often involve a conclusory leap from objective findings to subjective conclusions, without a clearly defined strategy of how the former led to the latter. The reliability and repeatability of final opinions are thus threatened by to the idiosyncrasies of the individual expert, making it difficult for factfinders to judge the accuracy of forensic medical expert opinions, primarily when they address complex issues of causality [65, 68, 69].

To improve the reliability and repeatability of expert opinions, we propose that all forensic medical practitioners should have an arsenal of various approaches to establish causation at their disposal in daily practice. This is not to say that all causal determinations require an added layer of complexity to increase their validity. In obvious cases (e.g. cause of death after a decapitation injury) common sense is a sufficient basis for a causal determination since it is incompatible with life. In more complex cases (e.g. blunt head trauma in an elderly individual with evidence of myocardial infarction and alcohol intoxication) a more robust and detailed approach is necessary in order to minimize the impact of speculation on a causal conclusion [70]. Because of a lack of tangibility inherent in causal analysis, even the term “cause” can have inconsistent meaning when used by different practitioners examining the same evidence. If a forensic medical practitioner concludes that “the cause of death was X”, is it meant that without X the death would not have occurred? Or that only once X was combined with Y and Z that X became a “cause” of death. Does the latter opinion mean that X by itself could not have caused the death, or that X, Y and Z were all independent explanatory causes for the death? In the example given above for the complex causation scenario, one expert may choose the head trauma as the cause of death, while another expert may choose the myocardial infarction, and a third expert may choose all three acting in concert. In other words, A causes B, if the probability of B given A is higher than the probability of B in the absence of A (everything else being constant) [15]. This causal inference based on counterfactual probability is suitable for use and readily applicable in legal proceedings because (1) it acknowledges the fact that a 100% degree of certainty for causality is unachievable, (2) it allows multiple causation (because a condition must only raise the probability of an event and does not have to be a sufficient cause for it, i.e. it is a contributory cause), (3) based on the ‘but-for’ concept, i.e. without the cause the outcome would not have occurred at all or at that particular time, and (4) it can be expressed as a probability of causation (PC) which can be calculated using epidemiological data from a population that is comparable to the affected individual [57, 71].

Currently, we are developing a causal analysis approach to be used in daily practice which is based on counterfactual inference. We hope that this new approach will provide simple guidance, perform adequately, and be scientifically sound, and yet still be practical and easy enough to communicate to medical laypersons.

## Conclusion

Currently, there exist several different approaches to medicolegal causal analysis, ranging from the simple, intuitive approach to complex calculations of the probability of causation based on Bayesian principles. Every approach has its own strengths and limitations, and thus forensic medical practitioners should employ the appropriate approach for different types of cases with varying degrees of complexity.

## Key points

1. Causal inference is a routine task in forensic medicine that is distinct from clinical diagnosis.
2. A variety of guides exist for causal inference, with various strengths and limitations.
3. A causal inference approach in forensic medicine is either intuitive or probabilistic.
4. There is no causal inference approach that can be applied universally.
5. Forensic medical practitioners must choose the appropriate approach for each case.
